# Biventricular function in exercise during autonomic (thoracic epidural) block

**DOI:** 10.1007/s00421-021-04631-6

**Published:** 2021-02-22

**Authors:** Jeroen Wink, Paul Steendijk, Roula Tsonaka, Rob B. P. de Wilde, Hans J. Friedericy, Jerry Braun, Bernadette Th. Veering, Leon P. H. J. Aarts, Patrick F. Wouters

**Affiliations:** 1 Department of Anesthesiology, Leiden University Medical Center, P.O. Box 9600, 2300 RC Leiden, The Netherlands; 2 Department of Cardiology, Leiden University Medical Center, Leiden, The Netherlands; 3 Department of Biomedical Data Sciences, Medical Statistics Section, Leiden University Medical Center, Leiden, The Netherlands; 4 Department of Intensive Care, Leiden University Medical Center, Leiden, The Netherlands; 5 Department of Cardio-Thoracic Surgery, Leiden University Medical Center, Leiden, The Netherlands; 6 Department of Anesthesia, University Hospitals Ghent, Ghent, Belgium

**Keywords:** Ventricular function, right, Ventricular function, left, Circulation, Anesthesia, Epidural, Nervous system, Autonomic, Exercise

## Abstract

**Background:**

Blockade of cardiac sympathetic fibers by thoracic epidural anesthesia (TEA) was previously shown to reduce right and left ventricular systolic function and effective pulmonary arterial elastance. At conditions of constant paced heart rate, cardiac output and systemic hemodynamics were unchanged. In this study, we further investigated the effect of cardiac sympathicolysis during physical stress and increased oxygen demand.

**Methods:**

In a cross-over design, 12 patients scheduled to undergo thoracic surgery performed dynamic ergometric exercise tests with and without TEA. Hemodynamics were monitored and biventricular function was measured by transthoracic two-dimensional and M-mode echocardiography, pulsed wave Doppler and tissue Doppler imaging.

**Results:**

TEA attenuated systolic RV function (TV Sʹ: − 21%, *P* < 0.001) and LV function (MV Sʹ: − 14%, *P* = 0.025), but biventricular diastolic function was not affected. HR (− 11%, *P* < 0.001), SVI (− 15%, *P* = 0.006), CI (− 21%, *P* < 0.001) and MAP (− 12%, *P* < 0.001) were decreased during TEA, but SVR was not affected. Exercise resulted in significant augmentation of systolic and diastolic biventricular function. During exercise HR, SVI, CI and MAP increased (respectively, + 86%, + 19%, + 124% and + 17%, all *P* < 0.001), whereas SVR decreased (− 49%, *P* < 0.001). No significant interactions between exercise and TEA were found, except for RPP (*P* = 0.024) and MV E DT (*P* = 0.035).

**Conclusion:**

Cardiac sympathetic blockade by TEA reduced LV and RV systolic function but did not significantly blunt exercise-induced increases in LV and RV function. These data indicate that additional mechanisms besides those controlled by the cardiac sympathetic nervous system are involved in the regulation of cardiac function during dynamic exercise.

*Trial registration* Clinical trial registration: Nederlands Trial Register, NTR 4880 http://www.trialregister.nl/trialreg/admin/rctview.asp?TC=4880.

## Introduction

Activation of the sympathetic nervous system is considered a key component in cardiovascular homeostasis (Breslow et al. 1989) and mobilization of cardiovascular reserve. As part of an integrated metabolic system, the ability of the heart to augment cardiac output (CO) is the main determinant of enhanced oxygen delivery during stress induced by exercise. Increments in CO are established by sympathetically mediated positive chronotropic and inotropic effects along with metabolism-induced reductions in systemic vascular resistance and enhanced muscle pump function. Thoracic epidural anesthesia (TEA) reduces sympathetic outflow to the heart and may induce substantial changes in heart rate and ventricular function, depending on the prevailing level of sympathetic tone. We recently demonstrated, using pressure–volume analysis, that TEA impairs right ventricular (RV) function without affecting CO (Wink et al. 2016). However, blockade of cardiac sympathetic innervation during exercise may reduce the degree with which the heart accelerates and enhances contraction. As such TEA provides a useful means for the study of sympathetic control mechanisms during stress. Previous studies that assessed the effects of β-blockers during exercise focused on general hemodynamics and left ventricular function. However, there is evidence that exercise-induced increases in CO lead to a greater load for the RV compared to the LV (La Gerche et al. 2017). The present study, therefore, was designed to evaluate the biventricular effects of TEA during dynamic ergometric exercise. LV and RV systolic and diastolic function was assessed using pulsed-wave tissue Doppler imaging (TDI).

The aims of the study were twofold. First, this study may provide additional insight into the role and effect size of the cardiac sympathetic nervous system on changes in biventricular function during exercise. Second, the study design using dynamic ergometric exercise may mimic hemodynamic changes and elevations of sympathetic tone as present during surgery and thus may be relevant for clinical applications of TEA. In general, cardiac function has been shown to be an important determinant of outcome in cardiothoracic surgery (Reed et al. 1996; Risum et al. 1996). From this perspective, TEA-induced decreases in cardiac reserve and alterations in circulatory control may potentially counteract the proclaimed beneficial effects in particular patient groups. The observation in several studies that use of TEA in high-risk patients is associated with worse cardiovascular outcome seems to support this theory (Leslie et al. 2013; Powell et al. 2011). The mechanisms behind TEA-associated cardiovascular problems are still poorly understood.

## Methods

The protocol of this study was reviewed and approved by the Committee on Medical Ethics of the Leiden University Medical Center, reg. no: P14.044, date: 07 Jan 2015 and registered (Nederlands Trial Register, NTR 4880). Between January 2015 and July 2017, patients above 18 years scheduled for thoracic surgery (full lateral thoracotomies or video-assisted thoracoscopic surgery/VATS) under TEA were asked to participate in this study and were enrolled after written informed consent. Patients with contra-indications for TEA, a history of coronary artery disease (CAD), ejection fraction < 40%, severe regurgitation or stenosis of a heart valve (grade 3 or 4), heart rhythm other than sinus rhythm, existence of diabetes mellitus, use of β-blockers or calcium-antagonists, pregnancy or lactation or participation in a trial on investigational drugs within 3 months prior to the study were excluded.

### Study design

The study was performed preoperatively in awake patients in the recovery room. A randomized cross-over design with two study arms was used to eliminate the effect of timing of the tests on treatment effects. An epidural catheter was placed on the day before surgery. Patients performed a supine exercise test on an ergometer at two distinct time points: the day before surgery (test period 1) and immediately before surgery (test period 2). In study arm A, patients received an epidural dose of 6 ml of NaCl 0.9% (control) in test period 1 and 6 ml of ropivacaine 0.75% (treatment) in test period 2. In study arm B, control and treatment were reversed. Patients were randomized to study arms A or B using a computer-generated randomization list (www.randomization.com).

**Fig. 1:**
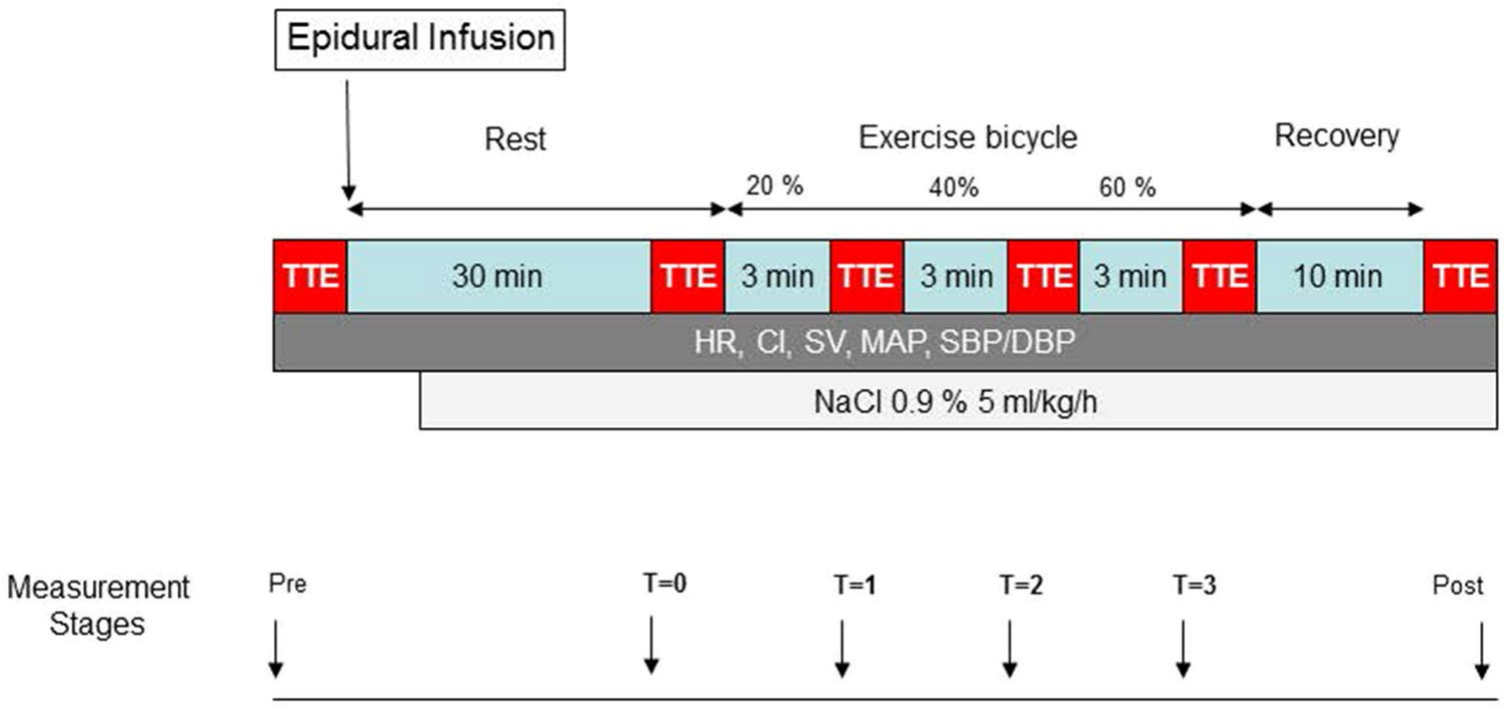
Measurement protocol executed during both test periods (Control and TEA). Hemodynamic and echocardiographic measurements were performed during different measurement stages: pre (pre-study), immediately before epidural injection of ropivacaine 0.75%/ NaCl 0.9%; T0, 30 min after epidural injection of ropivacaine 0.75%/ NaCl 0.9%; T1, after 3 min bicycling with 20% of maximal workload; T2, after 3 min bicycling with 40% of maximal workload; T3, after 3 min bicycling with 60% of maximal workload; post (post-study), after 10 min recovery of exercise test. *TTE* transthoracic echocardiography, *HR* heart rate, *CI* cardiac index, *SV* stroke volume, *MAP* mean arterial pressure, *SBP* systolic blood pressure, *DBP* diastolic blood pressure

During both test periods patients performed an incremental supine bicycle exercise test with hemodynamic and echocardiographic measurements at the predefined measurement stages as shown in Fig. 1.

Intravenous access was established and an infusion of NaCl 0.9% administered at a rate of 5 ml/kg/h starting with the epidural injection of ropivacaine 0.75% or NaCl 0.9%. Investigators were not blinded to epidural study medication during measurements and data acquisition. Evaluation of echocardiographic images and offline calculations were performed by a single investigator who was blinded to the treatment group, test period and stages of the exercise protocol.

### Premedication and preparations

Patients were allowed to have premedication with midazolam 5.0–7.5 mg orally 45 min before arrival at the recovery room. In case patients received premedication, it was administered before both test periods at equal doses.

### Monitoring and general hemodynamics

Heart rate (HR) and oxygen saturation were monitored continuously throughout the study, starting with pre-study measurements before the epidural injection. An arterial line 20 G was inserted after local infiltration with lidocaine 1% in the radial or brachial artery to continuously monitor mean arterial pressure (MAP), systolic blood pressure (SBP), and diastolic blood pressure (DBP) (Edward Lifesciences LLC, Irvine, Ca, USA). In addition, cardiac output (CO) and stroke volume (SV) were monitored using the Vigileo/FloTrac system (software version 4.00; Edwards Lifesciences, Irvine, CA). Systemic vascular resistance was calculated as SVR = 80. MAP/CO, thus neglecting central venous pressure (CVP). SV and CO were indexed to body surface area (SVI, CI). Rate pressure product (RPP) was used as an indicator of myocardial oxygen demand and was calculated as RPP = HR × SBP.

If SBP decreased more than 30% below the pre-anesthetic value or below 90 mmHg following the start of TEA, ephedrine 5 mg IV was given. Bradycardia (heart rate < 40 beats/min) was treated with atropine sulfate, 0.5 mg IV. All monitoring data were automatically recorded in an electronic database (Metavision).

### Thoracic epidural procedure

Insertion of the thoracic epidural catheter was performed at the T3–T4 level as described previously (Wink et al. 2016). After epidural catheterization, the patient was placed in the supine position on the bicycle and a pre-study TTE exam was performed.

After the pre-study TTE exam, patients received either 6 ml of NaCl 0.9% or 6 ml of ropivacaine 0.75% through their epidural catheter, depending on the study arm. Ropivacaine was first administered as a test dose of 3 ml 0.75% followed within 3 min by another 3 ml of ropivacaine in case there was no sign of intrathecal placement of the catheter. On completion of the measurements, patients returned to the ward. The epidural catheter was continuously flushed using NaCl 0.9% 2 ml/hr via a syringe pump to prevent obstruction.

### Treatment schedule

In a previous study, we demonstrated that thoracic epidural administration of 8 ml of ropivacaine 0.75% resulted in a rather large extension of sensory blockade (Wink et al. 2013). We, therefore, limited the epidural dose to 6 ml aiming at a level of epidural analgesia sufficient for surgery while avoiding extension of sensory and motor blockade to the legs.

### Assessments of analgesia

Analgesia was assessed bilaterally in the anterior axillary line over the chest and in the arms and legs by temperature discrimination using ice blocks. Results from both sides were averaged. All tests for analgesic and motor blocks were performed by the same investigator (JW). Motor block of the lower extremities was tested using the Bromage scale (0–3). Motor block of the upper extremities was tested by finger grip (C8/T1), hand flexion (C5/C6), and elbow flexion (ESSAM score) (Abd Elrazek et al. 1999). Maximum sensory and motor blockade was tested 30 min after epidural administration of the study component. The following parameters were assessed: highest and lowest dermatomal level of analgesia, maximum numbers of segments blocked and maximum score of motor block (Bromage scale and ESSAM score). Patients were withdrawn from the study in case motor blockade of the legs or a sensory block prevented them from performing the exercise.

### Echocardiography

Standard transthoracic (TTE) two-dimensional and M-mode echocardiography, pulsed wave Doppler and tissue Doppler imaging (TDI) examinations were performed with a Vivid 7 ultrasound machine (GE Healthcare, Hoevelaken, The Netherlands) equipped with a multifrequency phased-array transducer. All measurements were acquired from the AP 4CH view and performed by a board-certified echocardiographer.

Echocardiographic images were stored digitally for subsequent off-line analysis with EchoPac software (EchoPAC Dimension version 201; GE Vingmed Ultrasound AS, Horten, Norway). TDI images were acquired at frame rates above 150 Hz. All echocardiographic images were separated and filed according to the measurement stage and test period. Subsequently, these files were blinded for subject and measurement stage, then coded and digitally stored. The blinded files were presented in random order to the investigator responsible for the analysis of echocardiographic data (JW). For each workload, at least three beats at normal sinus rhythm were analyzed and averaged for all outcome variables.

### Left ventricular (LV) function

Pulsed wave TDI was used to quantify annular velocities of the mitral valve (MV) as peak systolic (MV Sʹ) and early and late diastolic mitral velocities at the lateral site sof the LV (resp. MV Eʹ and MV Aʹ).

In addition, pulsed wave Doppler was used to assess transmitral flow for peak velocity during early filling phase (MV E) and atrial contraction (MV A), the ratio of E to A velocities (MV E/A) and deceleration time (MV E DT).

### Right ventricular (RV) function

RV systolic function was assessed using Tricuspid Annular Plane Systolic Excursion (TAPSE). Pulsed wave TDI was used to quantify tricuspid valve annular velocity as peak systolic (TV Sʹ) and diastolic (TV Eʹ and TV Aʹ) velocities at the lateral site.

Pulsed wave Doppler was used to quantify transtricuspid flows as peak velocity during early filling phase (TV E), peak velocity during atrial contraction phase (TV A) and the ratio of E to A (TV E/A).

### Exercise test

The exercise test was performed with supine bicycle ergometry on a Cardiowise XRCISE Stress Echo ergometer (Cardiowise, Heilbronn, Germany), with the table inclined to 30–45° and tilted to 20–30° in the left lateral decubitus position. Individual maximal workload was determined using the formula:

$$\text{Max workload }\left( \text{Watt} \right)=\text{1}0\text{5 }-\text{ }\left( \text{2}.\text{525}\times \text{A} \right)\text{ }+\text{ }\left( 0.\text{8}0\text{83}\times \text{L} \right)\text{ }+\text{ }\left( 0.\text{575}\times \text{W} \right)$$

(A = age in years, L = length in cm, W = weight in kg).

For female patients, the max workload was adjusted to 80% of this calculated value.

Echocardiography (TTE) was performed according to a preset protocol with measurements repeated after 3 min of incrementally fixed workloads. The pedaling rate was kept constant using patient self-monitoring on a speed display at eye level. The initial workload was 20% of the maximal workload (T1) with subsequent increments towards 40% (T2) and 60% (T3). Patients were prompted to stop bicycling and start the recovery period in case of exhaustion, occurrence of chest pain and/or ST segment abnormalities on the ECG.

## Data analysis and sample size

### Objectives

Primary endpoints were TDI-based estimates of LV and RV systolic function (MV Sʹ and TV Sʹ, respectively). Additional echocardiographic data and hemodynamic data were considered secondary endpoints.

### Sample size

The sample size was calculated from expected changes in MV Sʹ during maximal exercise considering a 15% change in MV Sʹ, corresponding to a difference of 3.3 cm/s, as physiologically relevant. On the basis of a previous study (Bougault et al. 2008), we estimated an expected within-group standard deviation of 4.5 cm/s during peak exercise. A sample size of 17 patients would achieve 81% power to detect a significant change in MV Sʹ using the paired *t* test with a type I error of 0.05.

### Data analysis

All data are presented as means with range or standard deviation (SD), as appropriate. Outcome parameters were analyzed using a linear mixed effects model (LMM) to properly account for repeated measurements. In particular, a random intercept term was used. To capture the mean progression of each outcome parameter, we used an unstructured mean model with the following covariates: the main effect of exercise (taken as factor), the main effect of TEA, their interaction, and the main effect of period. Based on this model, several hypotheses were tested. First, we tested if the mean profiles were different between TEA and control during the stages T0–T3 (TEA effect). Second, we tested if mean outcome parameters were changed during T0–T3 (exercise effect). Third, we tested if the mean changes during the exercise levels T1–T3 vs T0 were statistically different between TEA and control (TEA–exercise interaction).

Normality of the residuals of the fitted models was checked and where appropriate the logarithmic transformation was applied to the relevant outcome parameters. All hypotheses were tested using the *F* test or the multivariate Wald test where appropriate. Results are reported via the corresponding *P* values and plots of the fitted mean profiles per group with standard deviation. All analyses were done in R (the R Development Core Team, www.R-project.org) using the packages lme 4 (Bates et al. 2015) and lmerTest (Alexandra Kuznetsova, Per Bruun Brockhoff and Rune Haubo Bojesen Christensen [2016]. lmerTest: Tests in Linear Mixed Effects Models. R package version 2.0–33. https://CRAN.R-project.org/package=lmerTest).

Statistical inference for the primary outcome variables MV Sʹ and TV Sʹ was corrected for multiple testing using the false discovery rate (FDR) method (Benjamini and Hochberg 1995). Other hemodynamic and echocardiographic parameters were not corrected for multiple testing. *P* values less than 0.05 were considered significant.

## Results

Fourteen patients were enrolled in this study. Two of them were not included in the final analysis because of failure of epidural placement (*N* = 1) or vasovagal collapse (*N* = 1) during epidural puncture. Demographics and data regarding neural blockade are presented in Table 1. Good-to-fair quality images were obtained in all patients. TDI analysis could not always be completed during the highest workload because of suboptimal image quality and/or fusion of E and A waves. However, MV Sʹ and RV Sʹ velocities were obtained at the highest workload in (92%) and 12 (100%) patients, respectively (T3).

**Table 1:** Patient characteristics and characteristics of neural blockade 30 min after epidural injection

Patient characteristics	*N* = 12
Age (years)	44 (18–68)
Gender (M/F)	7/5
ASA (I/II/III)	10/2/0
Height (cm)	182 (168–196)
Weight (kg)	83 (50–113)
Antihypertensive medication (yes/no)	0/12
Operation side (left/right/median)	6/5/1
Study arm (A/B)	5/7
Neural blockade 30 min after epidural administration of Ropivacaine 0.75%	
Highest level of analgesia (dermatome)	C5 (C3–T1)
Lowest level of analgesia (dermatome)	T8 (T6–L1)
Maximum number of spinal segments blocked	12 (9.0–17.5)
Maximum Bromage score (0–3)	0 (0.0–0.0)
Maximum ESSAM score (0–3)	0.3 (0.0–1.0)

Data are presented as mean (range)

*ASA* American Society of Anesthesiologists, *TEA* thoracic epidural anesthesia

### Conditions

All patients were able to complete the exercise test during control and TEA. The majority of patients reported a high level of fatigue during the maximal exercise level in both conditions, confirming the high intensity of exercise. None of the patients was treated with vasoactive medication during measurements and/or showed signs of coronary ischemia during exercise. The average total exercise time was comparable between the two sessions: control 22 min and TEA 23 min. Individual peak workloads ranged from 50 to 157 W. We tested if the TEA effects were different between the two study arms. There was no statistically significant carry-over effect for any of the outcome parameters and thus the period by treatment interaction term was excluded from the LMM.

### Measurements

The effects of TEA and exercise on echocardiographic and hemodynamic parameters are presented in Tables 2, 3, and 4. *P* values indicate statistical significance for TEA effects, exercise effects, and interaction effects between TEA and exercise.

**Table 2:** The effects of thoracic epidural anesthesia and exercise on global hemodynamics

	Conditions	Rest	Exercise stages			Effects		
		T0	T1	T2	T3	TEA	Exercise	Interaction
Global hemodynamics								
HR (beats/min)	Control TEA	63 (11) 61 (13)	82 (9) 76 (8)	99 (14) 88 (9)	117 (20) 106 (18)	*P* = 0.001	*P* < 0.001	*P* = 0.205
SBP (mmHg)	Control TEA	149 (18) 129 (22)	163 (24) 139 (27)	174 (28) 150 (28)	183 (28) 161 (25)	*P* < 0.001	*P* < 0.001	*P* = 0.931
DBP (mmHg)	Control TEA	69 (11) 62 (10)	67 (12) 62 (10)	68 (11) 63 (11)	72 (13) 68 (14)	*P* < 0.001	*P* = 0.002	*P* = 0.448
MAP (mmHg)	Control TEA	94 (12) 83 (12)	95 (14) 86 (12)	98 (13) 90 (12)	103 (15) 97 (14)	*P* < 0.001	*P* < 0.001	*P* = 0.417
RPP (mmHg/min.1000)	Control TEA	9.3 (1.7) 7.8 (1.9)	13.4 (2.2) 10.4 (1.9)	17.1 (3.3) 13.0 (2.2)	21.2 (3.9) 16.8 (2.7)	*P* < 0.001	*P* < 0.001	*P* = 0.024
CI (l/min/m^2^)	Control TEA	3.4 (0.9) 3.1 (0.7)	4.8 (1.5) 3.8 (0.9)	6.0 (1.9) 4.9 (1.1)	7.6 (2.8) 6.2 (1.2)	*P* < 0.001	*P* < 0.001	*P* = 0.215
SVI (ml/m^2^)	Control TEA	54 (11) 52 (13)	59 (18) 50 (10)	60 (14) 54 (12)	64 (17) 57 (12)	*P* = 0.006	*P* < 0.001	*P* = 0.672
SVR (dynes/s/cm^5^)	Control TEA	1155 (277) 1093 (222)	847 (259) 905 (193)	696 (209) 752 (164)	586 (181) 630 (113)	*P* = 0.389	*P* < 0.001	*P* = 0.535

Values at rest and during exercise are presented as mean (SD). Effects were determined by a linear mixed effects model (see Data analysis for details) and presented as *P* value. T0, 30 min after epidural injection of ropivacaine 0.75%/NaCl 0.9%; T1, after 3 min bicycling with 20% of maximal workload; T2, after 3 min bicycling with 40% of maximal workload; T3, after 3 min bicycling with 60% of maximal workload

*TEA* thoracic epidural anesthesia, *HR* heart rate, *SBP* systolic blood pressure, *DBP* diastolic blood pressure, *MAP* mean arterial pressure, *RPP* rate pressure product, *CI* cardiac index, *SVI* stroke volume index, *SVR* systemic vascular resistance

**Table 3:** The effects of thoracic epidural anesthesia and exercise on systolic and diastolic left ventricular function

	Conditions	Rest	Exercise stages			Effects		
		T0	T1	T2	T3	TEA	Exercise	Interaction
Systolic function								
MV Sʹ (cm/s)	Control TEA	10.8 (2.7) 10.6 (2.2)	13.0 (3.3) 12.4 (3.5)	16.1 (4.4) 13.8 (5.0)	16.9 (5.3) 15.7 (5.1)	*P* = 0.025	*P* < 0.001	*P* = 0.302
Diastolic function								
MV Eʹ (cm/s)	Control TEA	14.0 (4.0) 13.4 (4.1)	16.1 (3.3) 15.0 (3.3)	17.2 (4.3) 16.3 (3.0)	17.9 (4.3) 17.4 (4.1)	*P* = 0.470	*P* < 0.001	*P* = 0.956
MV Aʹ (cm/s)	Control TEA	8.6 (3.3) 8.3 (3.6)	10.3 (4.2) 10.2 (3.7)	12.7 (3.2) 10.3 (4.6)	12.7 (4.2) 14.8 (6.3)	*P* = 0.230	*P* < 0.001	*P* = 0.138
MV E (m/s)	Control TEA	0.76 (0.17) 0.76 (0.13)	0.89 (0.14) 0.87 (0.11)	0.98 (0.15) 0.96 (0.16)	1.09 (0.22) 1.13 (0.14)	*P* = 0.836	*P* < 0.001	*P* = 0.724
MV Dec T (ms)	Control TEA	169 (41) 186 (30)	178 (33) 153 (27)	163 (33) 139 (33)	134 (25) 141 (36)	*P* = 0.056	*P* < 0.001	*P* = 0.035
MV A (m/s)	Control TEA	0.64 (0.19) 0.55 (0.18)	0.74 (0.13) 0.68 (0.17)	0.81 (0.18) 0.72 (0.23)	0.85 (0.24) 0.81 (0.25)	*P* = 0.172	*P* < 0.001	*P* = 0.922
MV E/A	Control TEA	1.3 (0.36) 1.5 (0.54)	1.2 (0.35) 1.4 (0.33)	1.3 (0.29) 1.3 (0.32)	1.3 (0.42) 1.6 (0.64)	*P* = 0.039	*P* = 0.159	*P* = 0.701
MV E/Eʹ	Control TEA	5.6 (1.2) 6.0 (1.5)	5.7 (1.0) 6.0 (1.2)	6.0 (1.6) 6.0 (1.2)	6.0 (1.6) 6.7 (1.1)	*P* = 0.233	*P* < 0.001	*P* = 0.744
MV Eʹ/Aʹ	Control TEA	1.9 (1.0) 2.0 (1.2)	1.8 (0.6) 1.7 (0.8)	1.5 (0.6) 2.0 (1.1)	1.6 (0.8) 1.4 (0.7)	*P* = 0.345	*P* = 0.073	*P* = 0.231

Values at rest and during exercise are presented as mean (SD). Effects were determined by a linear mixed effects model (see Data analysis for details) and presented as *P* value. T0, 30 min after epidural injection of ropivacaine 0,75%/ NaCl 0,9%; T1, after 3 min bicycling with 20% of maximal workload; T2, after 3 min bicycling with 40% of maximal workload; T3, after 3 min bicycling with 60% of maximal workload

*TEA* thoracic epidural anesthesia, *MV S*ʹ peak systolic velocity of the mitral annulus, *MV E*ʹ early diastolic velocity of the mitral annulus, *MV A*ʹ late diastolic velocity of the mitral annulus, *MV E* peak mitral flow velocity during early filling phase, *MV Dec T* the time interval required for the E velocity to decline from its peak to the baseline, *MV A* peak mitral flow velocity during atrial contraction phase, *MV E/A* ratio of E to A, *MV E/E*ʹ ratio E to Eʹ, *MV E*ʹ*/A*ʹ the ratio of Eʹ to Aʹ

**Table 4:** The effects of thoracic epidural anesthesia and exercise on systolic and diastolic right ventricular function

	Conditions	Rest	Exercise stages			Effects		
		T0	T1	T2	T3	TEA	Exercise	Interaction
Systolic function								
TV Sʹ (cm/s)	Control TEA	14.3 (1.6) 12.3 (1.8)	16.1 (1.7) 14.4 (2.1)	20.4 (2.3) 16.1 (2.4)	21.5 (2.6) 19.0 (2.5)	*P* < 0.001	*P* < 0.001	*P* = 0.086
TAPSE (cm)	Control TEA	2.7 (0.4) 2.5 (0.3)	3.0 (0.4) 2.8 (0.2)	3.1 (0.5) 3.0 (0.5)	3.2 (0.6) 3.1 (0.6)	*P* = 0.097	*P* < 0.001	*P* = 0.719
Diastolic function								
TV Eʹ (cm/s)	Control TEA	14.9 (2.8) 14.8 (1.6)	17.9 (1.7) 16.0 (2.1)	21.3 (5.0) 20.8 (5.2)	22.5 (6.5) 21.5 (6.3)	*P* = 0.390	*P* < 0.001	*P* = 0.736
TV Aʹ (cm/s)	Control TEA	12.3 (3.6) 12.0 (4.2)	14.9 (4.7) 14.2 (4.6)	18.3 (5.9) 16.1 (4.3)	22.5 (6.0) 19.9 (6.2)	*P* = 0.342	*P* < 0.001	*P* = 0.721
TV E (cm/s)	Control TEA	0.56 (0.09) 0.57 (0.12)	0.61 (0.13) 0.61 (0.09)	0.73 (0.18) 0.80 (0.15)	0.81 (0.15) 0.82 (0.26)	*P* = 0.639	*P* < 0.001	*P* = 0.672
TV Dec T (ms)	Control TEA	222 (68) 218 (55)	169 (68) 176 (57)	138 (33) 143 (34)	128 (44) 124 (31)	*P* = 0.982	*P* < 0.001	*P* = 0.948
TV A (cm/s)	Control TEA	0.37 (0.10) 0.33 (0.08)	0.55 (0.11) 0.48 (0.15)	0.62 (0.14) 0.57 (0.14)	0.77 (0.15) 0.61 (0.18)	*P* = 0.005	*P* < 0.001	*P* = 0.330
TV E/A	Control TEA	1.6 (0.5) 1.8 (0.6)	1.2 (0.4) 1.4 (0.5)	1.2 (0.3) 1.6 (0.6)	1.1 (0.3) 1.3 (0.6)	*P* = 0.011	*P* = 0.004	*P* = 0.896
TV E/Eʹ	Control TEA	3.9 (1.2) 3.8 (0.7)	3.4 (0.8) 3.9 (0.9)	3.6 (1.0) 3.9 (0.7)	4.0 (1.1) 3.7 (1.0)	*P* = 0.368	*P* = 0.109	*P* = 0.391
TV Eʹ/Aʹ	Control TEA	1.4 (0.6) 1.4 (0.6)	1.4 (0.8) 1.2 (0.4)	1.4 (0.9) 1.4 (0.6)	1.0 (0.3) 1.1 (0.3)	*P* = 0.891	*P* = 0.230	*P* = 0.787

Values at rest and during exercise are presented as mean (SD). Effects were determined by a linear mixed effects model (see Data analysis for details) and presented as *P* value. T0, 30 min after epidural injection of ropivacaine 0.75% / NaCl 0.9%; T1, after 3 min bicycling with 20% of maximal workload; T2, after 3 min bicycling with 40% of maximal workload; T3, after 3 min bicycling with 60% of maximal workload

*TEA* thoracic epidural anesthesia, *TV S*ʹ peak systolic velocity of the tricuspid annulus, *TAPSE* tricuspid annular plane systolic excursion, *TV E*ʹ early diastolic velocity of the tricuspid annulus, *TV A*ʹ late diastolic velocity of the tricuspid annulus, *TV E* peak tricuspid flow velocity during early filling phase, *TV Dec T* the time interval required for the E velocity to decline from its peak to the baseline, *TV A* peak tricuspid flow velocity during atrial contraction phase, *TV E/A* ratio of E to A, *TV E/E*ʹ ratio E to Eʹ, *TV E*ʹ*/A*ʹ the ratio of E’ to A’

### General hemodynamics (Table 2 and Fig. 2)

**Fig. 2:**
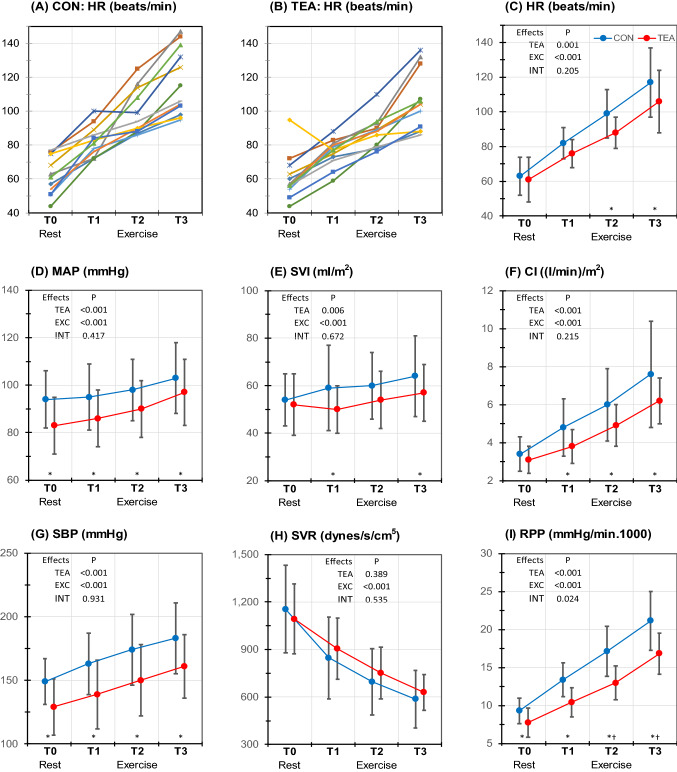
Individual and mean (SD) hemodynamic values at different measurement stages (measurement stages, see Fig. 1). Spaghetti plot of individual heart rates at control condition (a) and TEA (b); mean values of HR (c), MAP (d), SVI (e), CI (f), SBP (g), SVR (h) and RPP (i) during control (blue symbols) and TEA (red symbols). *P* values are presented for the overall effects of thoracic epidural anesthesia (TEA), exercise (EXC) and interaction effects between exercise and TEA (INT). In case of significant overall effects, the specific time points (T0–T3) at which significance was reached are indicated with * for a significant TEA effect and † for a significant TEA–exercise interaction effect. *CON* control, *HR* heart rate, *SVI* stroke volume index, *CI* cardiac index, *SBP* systolic blood pressure, *SVR* systemic vascular resistance, *RPP* rate pressure product

Compared to control, TEA significantly reduced all hemodynamic parameters except SVR. Maximal reductions were − 11% for HR, − 24% for RPP, − 15% for SVI and − 21% for CI.

During exercise HR, SVI and CI increased (maximal + 86%, + 19% and + 124% versus T0, respectively). MAP, SBP and DBP also increased (+ 17%, + 25% and + 10%, respectively) despite significant reductions in SVR (− 49%) in both conditions.

No significant interactions between TEA and exercise were found, except for RPP (*P* = 0.024).

### Left ventricular (LV) function (Table 3 and Fig. 3)

**Fig. 3:**
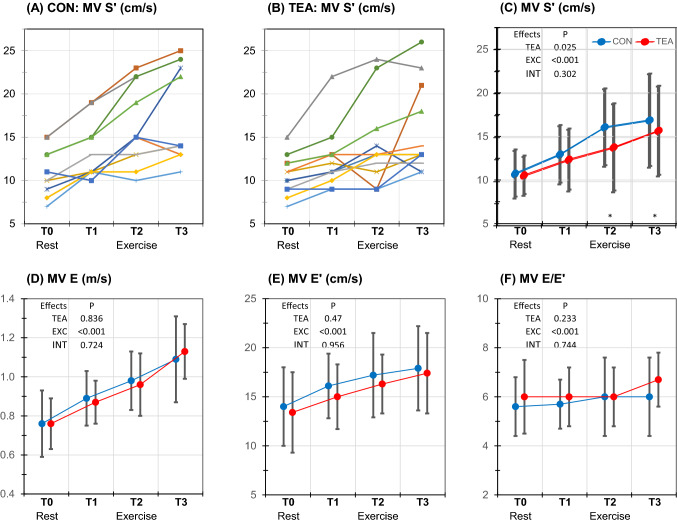
Individual and mean (SD) echocardiographic values for left ventricular function at different measurement stages (measurement stages, see Fig. 1). a Spaghetti plot of individual MV Sʹ values at control condition (a) and TEA (b); mean values of MV Sʹ (c), MV E (d), MV Eʹ (e) and MV E/Eʹ (F) during control (blue symbols) and TEA (red symbols). *P* values are presented for the overall effects of thoracic epidural anesthesia (TEA), exercise (EXC) and interaction effects between exercise and TEA (INT). In case of significant overall effects, the specific time points (T0-T3) at which significance was reached are indicated with * for a significant TEA effect and † for a significant TEA–exercise interaction effect. *CON* control, *MV S*ʹ peak systolic velocity of the mitral annulus, *MV E* peak mitral flow velocity during early filling phase, *MV E*ʹ early diastolic velocity of the mitral annulus, *MV E/E*ʹ ratio E to Eʹ

#### Systolic LV function

TEA induced a significant decrease in LV systolic function, reflected by decreases in MV Sʹ (− 14%).

Exercise resulted in increases in MV Sʹ (maximal + 56%).

There were no significant interaction effects between TEA and exercise.

#### Diastolic LV function

TEA had no effect on MV Eʹ, MV Aʹ, MV E and MV A, but there was a small increase in MV E/A (*P* = 0.039).

Exercise augmented MV Eʹ (maximal + 30%), MV Aʹ (+ 78%) as well as MV E (+ 49%) and MV A (+ 47%). There was a small but significant increase in E/Eʹ (+ 12%) while MV E/A remained unchanged from resting values.

TEA was associated with a steeper decline in MV E DT (*P* = 0.035) during exercise. No other interactions were found.

### Right ventricular (RV) function (Table 4 and Fig. 4)

**Fig. 4:**
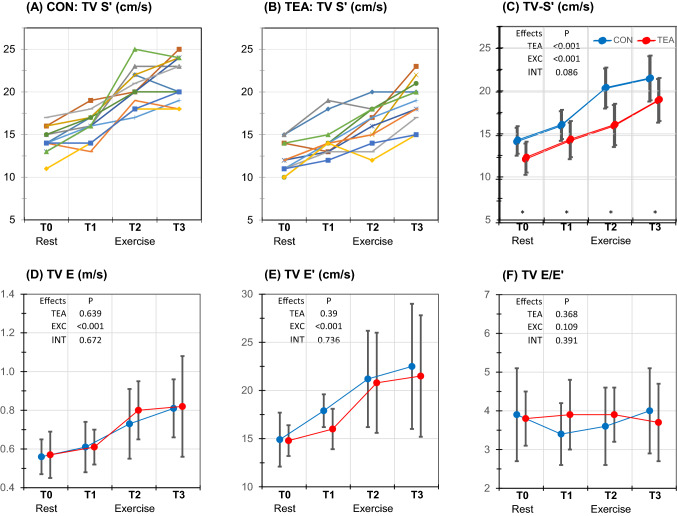
Individual and mean (SD) echocardiographic values for right ventricular function at different measurement stages (measurement stages, see Fig. 1). a Spaghetti plot of individual TV Sʹ values at control condition (a) and TEA (b); mean values of TV Sʹ (c), TV E (d), TV Eʹ (e) and TV E/Eʹ (f) during control (blue symbols) and TEA (red symbols). *P* values are presented for the overall effects of thoracic epidural anesthesia (TEA), exercise (EXC) and interaction effects between exercise and TEA (INT). In case of significant overall effects, the specific time points (T0-T3) at which significance was reached are indicated with * for a significant TEA effect and † for a significant TEA-exercise interaction effect. *CON* control, *TV S*ʹ peak systolic velocity of the tricuspid annulus, *TV E* peak tricuspid flow velocity during early filling phase, *TV E*ʹ early diastolic velocity of the tricuspid annulus, *TV E/E*ʹ ratio E to Eʹ

#### Systolic RV function

TEA decreased TV Sʹ (− 21%) but did not significantly affect TAPSE.

Exercise significantly increased TV Sʹ (+ 54%) and TAPSE (+ 24%).

Data suggested an interaction TEA-Exercise effect for TV Sʹ, with the effect of TEA being larger at higher levels of exercise. However, after correcting for multiple testing significance was lost (*P* = 0.086).

#### Diastolic RV function

TEA significantly decreased TV A (− 21%) and increased TV E/A (+ 33%).

Exercise increased TV Eʹ (+ 51%), TV Aʹ (+ 83%), TV E (+ 45%) and TV A (+ 108%) and decreased TV E DT (− 43%) and TV E/A (− 31%). TV E/Eʹ and TV Eʹ/Aʹ were not affected.

No interaction TEA-exercise effects were found.

## Discussion

The present study shows that cardiac sympathetic blockade following thoracic epidural anesthesia (TEA) causes a biventricular decrease in systolic function but does not abolish the hemodynamic response to exercise in patients scheduled to undergo thoracic surgery. Stepwise increases in bicycle exercise produced a proportional increase in biventricular systolic function and cardiac output, while systemic vascular resistance decreased. This pattern was not altered by the presence of TEA, although absolute values for cardiac output and ventricular function were always lower with than without TEA at each level of exercise. Our data suggest that TEA may affect cardiovascular reserve; however, mechanisms other than cardiac sympathetic reflexes play a dominant role in the cardiovascular adaptation to exercise.

Previous studies have shown that TEA decreases LV and RV function (Goertz et al. 1993; Missant et al. 2010; Wink et al. 2016, 2014). Indeed, both ventricles are densely innervated by sympathetic nerves (Ito and Zipes 1994; Momose et al. 2001) which originate from the first to the fifth thoracic spinal cord segment. When TEA is used as an analgesic technique in patients undergoing thoracic and high abdominal surgery, the epidurally injected local anesthetics also block cardiac sympathetic nerves (Wink et al. 2020). Interestingly, the mild cardiodepressant effects of TEA do not produce hemodynamic instability in patients at rest or under general anesthesia. In fact, TEA is considered beneficial in subjects with ischemic heart disease who benefit from the associated reduction in myocardial oxygen demands.

While the functional integrity of the cardiac sympathetic nervous system may not be essential when metabolic demands are low, it is considered to play a key role in the hemodynamic adaptation to exercise, stress and cardiovascular disruption (Bengel et al. 2001; Kirno et al. 1994). After heart transplantation, patients with and without sympathetic reinnervation have a similar cardiac performance at rest, but the former group showed better chronotropic and inotropic responses as well as improved performance during exercise (Bengel et al. 2001). Since major surgery is frequently associated with hemodynamic challenges, the use of TEA could, at least in theory, interfere with the essential cardiovascular adaptive responses. We tested this hypothesis by subjecting patients to dynamic exercise in the presence and absence of TEA. The elevated sympathetic tone created with bicycle exercise appears to mimic surgical stress encountered in the perioperative period: both conditions are reported to lead to three- to fourfold increases in plasma norepinephrine levels (Schwab et al. 1992; Yokoyama et al. 2005). Exercise was shown to result in up to 100% increases in the contractile state of the left and right ventricles (Bougault et al. 2008; Chia et al. 2015; Reuss et al. 2005). Two previous studies examined the general hemodynamic effects of TEA during bicycle exercise, but they did not quantify cardiac function. In these studies, TEA produced limited effects on HR, CO, MAP and oxygen extraction (Ottesen 1978; Wattwil et al. 1985), in line with our findings.

We used TDI to quantify ventricular pump function because this technique has been extensively validated in clinical practice. TDI-derived variables were demonstrated to be robust and reproducible determinants of cardiac function during exercise (Bougault et al. 2008; Chia et al. 2015; Reuss et al. 2005). In our study, TEA decreased peak systolic velocities (Sʹ) of both the mitral and tricuspid valvular annulus and this effect was more pronounced for the right ventricle. Changes in peak systolic velocities of the atrioventricular annulus not only correlate directly with contractile performance of the heart but also show an inverse relationship with changes in ventricular afterload. Since systemic vascular resistance did not change with TEA, the small but consistent decrease in peak systolic velocities of the mitral annulus can be attributed primarily to a decreased LV function. Unfortunately, pulmonary vascular resistance was not quantified in our study, so similar inferences cannot be made for the RV. Non-invasive estimation of pulmonary arterial pressures is routinely performed in clinical practice, using Doppler interrogation of a tricuspid regurgitant jet, but we found these measurements cumbersome, time-consuming and unreliable when patients were exercising. Previous studies addressing the effect of TEA on the pulmonary vascular system have produced conflicting results. Brimioulle et al. showed increased pulmonary tone after extensive TEA in anesthetized animals (Brimioulle et al. 1997). This effect was blocked with adrenergic antagonists, however, which is difficult to explain in the framework of a direct TEA-induced sympatholysis. Other studies found that TEA had no effect on the pulmonary vasculature or even decreased pulmonary tone. We recently reported that TEA caused a mild decrease in pulmonary vascular elastance, based on P–V loop analysis in patients undergoing thoracic surgery (Wink et al. 2016). In that study, we used load-independent indices of contractility to assess cardiac function and also found a direct depressant effect of TEA on the RV.

The pulmonary vascular bed has a limited vasodilator reserve (Dawson 1984; Naeije et al. 2013) and the capacity to accommodate for exercise-induced increases in cardiac output is substantially lower in the pulmonary than in the systemic vascular bed (La Gerche and Claessen 2015). As a consequence, exercise causes an increase in the PAP-to-MAP ratio (Ottesen 1978) and induces a higher hemodynamic load on the RV than on the LV (La Gerche et al. 2017). Progressive increases in left atrial pressures (Kovacs et al. 2009; Reeves et al. 1990) may further contribute to the rise in pulmonary vascular pressures and RV afterload caused by exercise. Interventricular differences in hemodynamic load and the dependency on sympathetic reflexes to mobilize inotropic reserve could explain the observation that TEA had a more pronounced effect on RV function.

From a clinical perspective, our findings are reassuring: TEA exerted only mild effects on the hemodynamic response to exercise. It has been suggested earlier that TEA attenuates sympathetic neural transmission rather than causing complete blockade (Malmqvist et al. 1989; Stevens et al. 1995). In addition, catecholamine release from the adrenal glands was probably not inhibited by TEA in our study as the lower border of analgesia was spinal level T8 on average. However, β-blockers were reported to reduce HR, SV and CO during exercise by only 15–25% (Clifton et al. 1990; Epstein et al. 1965; Fleg et al. 1994; Gullestad et al. 1996; Horwitz et al. 1974). These results are similar to our results with TEA and highlight the importance of other, non-adrenergic mechanisms driving the cardiovascular adaptation to exercise.

We acknowledge that the present study has limitations which need consideration when interpreting the results. First, we selected a randomized cross-over design with two study arms to increase the statistical power, allowing a relatively small patient sample. The design also aimed to eliminate the effect of timing of the tests on treatment effects: because of the large time interval between the two test periods, we assumed no carry-over effects related to infusion of local anesthetics or the repeated exercise test. This was confirmed statistically for all outcomes analyzed. Despite this, the sample size was relatively small which may have limited the power to detect TEA-exercise interaction effects. Second, we used pulsed wave TDI with high sampling frequency (> 150 Hz) to obtain reliable myocardial velocities during exercise. However, this frame rate may still be insufficient to capture peak velocities at high HR; hence, systematic underestimation of peak velocities at higher levels of exercise is possible. Third, all patients included in this study had normal cardiovascular function. The findings should, therefore, not be transposed to high-risk populations. Finally, cardiovascular stress induced by dynamic exercise may be relevant for a number of perioperative conditions but clearly differs from other frequently encountered hemodynamic challenges such as hypovolemia and vascular clamping.

## Conclusion

Cardiac sympatholysis with TEA consistently reduced LV and RV systolic function without affecting diastolic biventricular function. Importantly, TEA did not eliminate the cardiovascular response to exercise and augmentation of LV and RV function was largely preserved. TEA affects cardiovascular reserve, however, mechanisms other than those involving cardiac sympathetic reflexes play a dominant role in the cardiovascular adaptation to exercise.

## Abbreviations

CAD: Coronary artery disease
CI: Cardiac index
CO: Cardiac output
CVP: Central venous pressure
DAP: Diastolic blood pressure
HR: Heart rate
IVA: Isovolumetric acceleration
LMM: Linear mixed effects model
LV: Left ventricle
MAP: Mean arterial pressure
MV: Mitral valve
MV A: Peak transmitral velocity during atrial contraction
MV Aʹ: Peak late diastolic mitral valve velocity at the lateral site of the LV
MV E: Peak transmitral velocity during the early filling phase
MV Eʹ: Peak early diastolic mitral valve velocity at the lateral site of the LV
MV E/A: Ratio of mitral E to A velocities
MV E DT: Time interval required for the E velocity to decline from its peak to the baseline
MV Sʹ: Peak systolic mitral valve velocity at the lateral site of the LV
PAP: Pulmonary artery pressure
PVR: Pulmonary vascular resistance
RPP: Rate pressure product
RV: Right ventricle
SBP: Systolic blood pressure
SD: Standard deviation
SV: Stroke volume
SVI: Stroke volume index
SVR: Systemic vascular resistance
TAPSE: Tricuspid annular plane systolic excursion
TEA: Thoracic epidural anesthesia
TDI: Tissue Doppler imaging
TTE: Transthoracic echocardiography
TV A: Peak transtricuspid velocity during atrial contraction
TV Aʹ: Peak late diastolic tricuspid valve velocity at the lateral site of the RV
TV E: Peak transtricuspid velocity during early filling phase
TV Eʹ: Peak early diastolic tricuspid valve velocity at the lateral site of the RV
TV E/A: Ratio of tricuspid E to A velocities
TV Sʹ: Peak systolic tricuspid valve velocity at the lateral site of the RV
VATS: Video-assisted thoracoscopic surgery
